# Endoscopic Treatment of Zenker's Diverticulum: Comparable Treatment Outcomes in Treatment-Naïve and Pretreated Patients

**DOI:** 10.1155/2021/9237617

**Published:** 2021-03-16

**Authors:** Johannes Manzeneder, Christoph Römmele, Carolin Manzeneder, Alanna Ebigbo, Helmut Messmann, Stefan Karl Goelder

**Affiliations:** Department of Internal Medicine III at the University Hospital Augsburg, Germany

## Abstract

**Background and Aims:**

Flexible endoscopic treatment plays an important role in the treatment of Zenker's diverticulum (ZD). This study analyzes long-term symptom control and the rate of adverse events in treatment-naïve patients and patients with recurrence, using the stag beetle knife junior (sb knife jr).

**Methods:**

From August 2013 to May 2019, 100 patients with symptomatic ZD were treated with flexible endoscopy using the sb knife jr. Before treatment, as well as 1 and 6 months afterwards, symptoms were obtained by a nine-point questionnaire, with symptoms weighted from 0 to 4.

**Results:**

Overall, 126 interventions were performed. The median follow-up period was 41 months (range 7-74). For the three most frequent symptoms, regurgitation, dysphagia, and dry cough, a significant reduction of the mean score could be achieved, from 2.85/3.45/2.85 before the initial treatment to 0.56/1.09/0.98 6 months later. 17 patients were retreated because of recurrence. Out of these, 12 patients underwent a 2^nd^, 4 patients a 3^rd^, and 1 patient a 4^th^ session, respectively. The mean dysphagia score for successfully treated patients could be reduced from initially 2.34 to 0.49/0.33/0.67 after the 1^st^/2^nd^/3^rd^ session, the frequency of dysphagia from 3.45 to 0.92/1.00/1.33, and the score for regurgitations from 2.85 to 0.35/1.00/0.67. In first-line treatment, as well as in retreatment, no severe adverse event occurred.

**Conclusion:**

Patients with ZD can be treated safely and effectively with the sb knife jr. Retreatment leads to equal symptom relief as compared to a successful first-line treatment and is not associated with a higher rate of adverse events.

## 1. Introduction

Zenker's diverticulum (ZD) is a rare disease of the laryngopharynx that appears mainly in elderly people. It emerges in a weak part of the inferior pharyngeal constrictor muscle called the triangle of Killian which is located superior to the upper esophageal sphincter [1, 2]. Patients with ZD primarily suffer from dysphagia and regurgitations. But there are a number of other symptoms such as halitosis, aspiration, dry cough, and vomiting in varying frequency and intensity associated with ZD [1, 3].

For a long time, the treatment had been either surgical or peroral with a rigid endoscope mostly done by Ear-Nose-Throat (ENT) physicians. In the midnineties, an approach with flexible endoscopy was presented for the first time [4, 5]. Since then, a huge variety of techniques and devices have been introduced [6]. Currently, flexible endoscopy plays an important role in the treatment of ZD. Basically, endoscopic approaches have in common the incision of the diverticular bridge with varying success and recurrence rates. However, the recurrence rate remains an issue of controversy, especially when comparing endoscopic and surgical techniques [1, 6, 7].

The aim of this study is to investigate symptom control and the rate of adverse events in flexible endoscopic treatment of ZD with the stag beetle knife junior (sb knife jr) in first-line therapy as well as in the therapy of symptomatic recurrence.

## 2. Methods

### 2.1. Patients

Data from patients with symptomatic ZD who were treated at the Department of Internal Medicine III at the University Hospital Augsburg, Germany, from August 2013 to May 2019 were evaluated. All patients were treated with flexible endoscopy and the sb knife jr as the cutting device. Patients who had had a previous treatment, either surgical or endoscopic, were excluded.

### 2.2. Endoscopic Procedure and Devices

All interventions were done by two experienced endoscopists (H.M., S.K.G.). A single treatment protocol was implemented.

**Figure 1:**
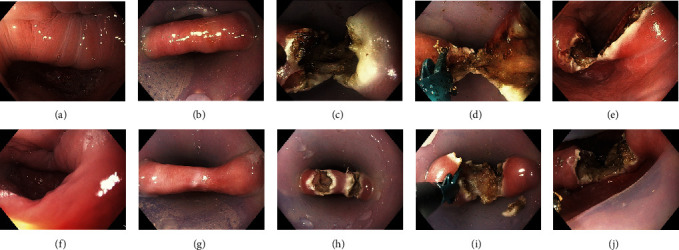
(a) Symptomatic ZD of a 73-year-old male patient. (b) Fixation of the diverticulum with an overtube. (c) Incision of the mucosa and the upper muscular fibers of the diverticular bridge. (d) Cutting down the diverticular bridge with the sb knife jr. (e) Final result after the first session. (f–j) Second session because of recurrence 16 months later.

On the day prior to the intervention, a gastroscopy was performed to clean the diverticulum of food remnants, to measure the size, and to inspect the diverticulum in order to exclude patients with a candida infection. Diverticulotomy was performed in deep sedation with midazolam, pethidine, and propofol. First, a soft diverticuloscope (ZD overtube, ZDO-22/33 Cook Medical, Limerick, Ireland) was placed to stretch the diverticular bridge between the esophagus and the diverticular lumen. The endoscope (GIF-HQ190, Olympus Europa, Hamburg, Germany) was inserted, and the mucomyotomy was done with the sb knife jr (Sumitomo Bakelite, Tokyo, Japan). This is a scissors-shaped device with an opening width of 3.5 mm that can be rotated by 360 degrees. Additional to electrical cutting, the device is able to simultaneously compress the tissue. Due to the mechanical effect, the directly adjacent tissue is more strongly bonded together (Figure 1). The electrosurgical current was generated by the VIO 300 unit (Endocut Q Effect 1, soft coagulation 40 W, Erbe, Tübingen, Germany).

The technique was modified in the course of the study; initially, only one incision was done in the middle of the diverticular bridge. Later, two incisions were made and the part in-between was resected with a snare (double incision and snare resection (DISR)) in order to get a broader incision of the diverticular bridge. The incision was then continued in the middle to the base of the diverticulum [8]. Regardless of the technique used, the aim was to completely cut the fibers of the upper esophageal sphincter. Also, the treatment of recurrences was done using these described techniques.

Each patient received a single dose of antibiotics (2 g ceftriaxone) during the intervention. Clipping of the bottom of the incision line was not done routinely. Prophylactic clipping to prevent delayed bleeding or perforation was performed in some cases, based on the judgment of the endoscopist.

Recurrence was defined as a relapse of symptoms with substantial limitations in a patient's quality of life. Adverse events were classified according to the American Society for Gastrointestinal Endoscopy (ASGE) [9].

### 2.3. Postprocedural Management and Follow-Up

On the day after the intervention, a contrast swallow was performed. If there was no evidence of perforation, the transition to a soft diet for five days was commenced.

The symptoms of the patients were recorded before treatment as well as one and six months after the intervention by a questionnaire developed in our clinic [3, 10]. The questionnaire contains nine points: Dakkak and Bennett's dysphagia score [11]; the frequency of dysphagia, odynophagia, regurgitation, vomiting, dry cough, halitosis, and nocturnal awakening due to Zenker-related symptoms; and the general condition of the patient including body weight, duration of symptoms, and weight loss. The symptoms were registered on an ordinal scale with values from zero to four (Table 1). All patients were informed about the possibility of readmission in case of recurring symptoms. Additionally, patients who stated a high point value (>12) in the questionnaire six months after the initial intervention were called to evaluate whether further treatment was necessary.

### 2.4. Statistics

The evaluation was performed using Microsoft Excel. Data were stated as mean, median, and standard deviation. For statistical analysis, different *t*-tests and chi-squared tests were used. Statistical significance was assumed at a *p* value of <0.05.

### 2.5. Ethical Considerations

All patients gave written consent to the intervention. The study was conducted in accordance with the Declaration of Helsinki. The study protocol was approved by the institutional review board (Reference number: BKF 2018-19).

## 3. Results

### 3.1. Patients and Symptoms

Overall, 100 patients with symptomatic ZD were treated. 36 (36%) of them were female, and the median age was 71 years (range 42-92 years). All diverticula were stadium III or IV according to the radiological classification of Brombart [12]. The median size was 20 mm (range 10-45 mm). The median body mass index (BMI) was 26.1 kg/m^2^ (range 17.3-38.0 kg/m^2^), and the mean weight loss before treatment was 2.3 kg (range 0-20 kg).

In total, 126 interventions were performed (1.26 sessions/patient). Of these, 60 (47.6%) were done in the modified DISR technique.

In three cases, cutting off the diverticular bridge was not completed in the first session and a second intervention had to be planned. The reason for this was a cyst in the diverticular bridge, a large asymmetric diverticulum, and, in the third case, suspicion of a small perforation, which was not confirmed afterwards.

Clips were used in 21 interventions (range 1-5). In most cases, the clipping was done only for prophylactic reasons (*n* = 14, 66.7%). Clips were applied four times (19.0%) for bleeding control and in three cases (14.3%) because of suspected perforation.

The rate of return of the questionnaire was 91% after one month and 90% after six months. The median follow-up was 41 months (range 7-74) (Table 2).

The patients showed a wide variety of symptoms (Table 3). The most frequent symptoms were regurgitation, dysphagia, and dry cough (mean score before intervention 2.85/3.45/2.85). Permanent dysphagia occurred in 62% of patients and in 27% more than once a week. After treatment, there was a significant reduction of symptoms. One month after the intervention, the mean score for regurgitations was 0.30 (*p* < 0.001, compared to the value prior to the treatment), for dysphagia 0.62 (*p* < 0.001), and for dry cough 0.78 (*p* < 0.001). After one month, the majority of patients (64%) had no dysphagia. Also, six months after the intervention, a significant reduction of symptoms could be reported (median score for regurgitations 0.56, *p* < 0.001; for dysphagia 1.09, *p* < 0.001; and for dry cough 0.98, *p* < 0.001).

### 3.2. Adverse Events

Intraprocedural bleeding occurred in 16 interventions (12.7%). Most cases (15) were stopped by coagulation with the sb knife jr or by a hemostatic grasper (Coagrasper Hemostatic Forceps FD-411 QR, Olympus Europa, Hamburg, Germany). In four cases, clips (range 1-2, Olympus Medical Systems Corp., Tokyo, Japan) were used. In three cases, a combination of a hemostatic grasper and hemoclips was applied. Termination of the procedure due to intraprocedural bleeding was not necessary since all bleeding cases could be treated successfully. There was no case of delayed bleeding. Procedures performed using the DISR technique showed a slightly lower bleeding rate, but this was not significant (DISR 12.2%, single incision 18.6%, and *p* = 0.39).

In four cases, a perforation was suspected during the intervention. Of these, one intervention had to be stopped prematurely. In five other cases, contrast swallow after the treatment showed a small perforation. In case a perforation was suspected, nil diet and clinical observation were extended and antibiotics were given for several days. But in all these cases, the further clinical course was uneventful. Emphysema was not observed in any of the patients.

Three patients suffered severe or prolonged postprocedural pain. One patient was monitored overnight in the intensive care unit to rule out serious adverse events as a reason for his pain. All these patients were managed conservatively.

Another patient developed a hemodynamically relevant tachycardia (focal atrial tachycardia) after the intervention, most probably because she had not taken her antiarrhythmic medication prior to the intervention. She spent one night in the intensive care unit and was treated with amiodarone.

In all 126 interventions, no severe adverse event was observed. There was no case of mediastinitis or abscess.

### 3.3. Recurrence

**Figure 2:**
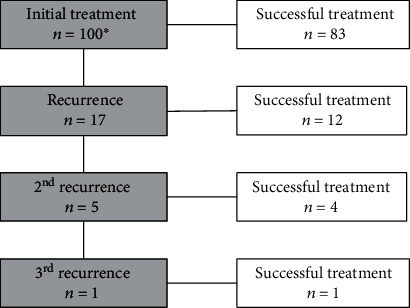
Flow chart of patients with recurrence. Recurrence: recurring symptoms after a temporary improvement. ^∗^103 sessions in 100 patients due to three two-stage treatments.

After initial treatment, 17 patients (17%) developed a recurrence (Figure 2). Nine (9%) recurrences occurred within the first six months after treatment, and eight (8%) later than six months. Five patients needed a third session and one a fourth.

The demographic and clinical characteristics of patients who developed a recurrence did not differ from patients without recurrence (Table 4). Furthermore, the size of the diverticulum did not correlate with the risk of recurrence (*p* = 0.26). The median diverticular size in patients with recurrence was 15 mm (range 5-35 mm). Considering the recurrence rate, there was no significant difference between DISR and single incision technique (DISR 17.1%, single incision 16.9%, and *p* = 0.99). Also, the use of clips during the first intervention had no influence on the recurrence rate (clips used 10.5%, no clips 18.5%, and *p* = 0.40).

**Figure 3:**
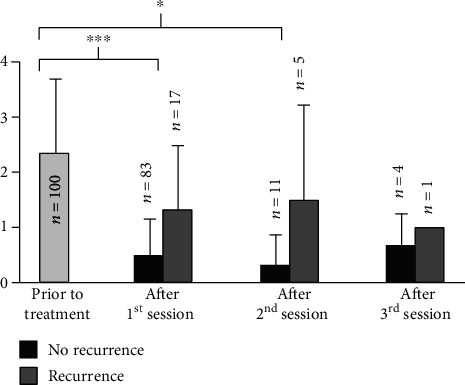
Dysphagia score of patients with and without recurrence after each session. Mean dysphagia score prior to initial treatment and six months after each session. Dysphagia score: 0: no dysphagia, 1: solid food, 2: soft food, 3: fluids, and 4: aphagia. ^∗∗∗^*t*-test significance *p* < 0.001, ^∗^*t*-test significance *p* < 0.05.

After retreatment, symptom scores of patients with recurrence could be reduced to a level comparable to patients who were recurrence-free after one intervention. Dakkak and Bennett's dysphagia score was reduced from 2.34 to 0.49 (*p* < 0.001) in patients without recurrence. Those patients who developed a recurrence after the initial treatment had an average score of 1.31 six months after the first intervention. Six months after the second treatment, the score was 0.33 (*p* = 0.01) for patients with no further recurrence and 1.50 for patients with a second recurrence. Besides one patient, all remaining patients could be treated successfully in the third session. Their dysphagia score was 0.67 six months after the third intervention which is comparable to the values of those patients who did not develop a further recurrence after the first or second treatment. Due to the small number of patients in this group (available data from three out of four patients), a reasonable statistical calculation is not possible in that case (Figure 3).

The same effect was seen for the frequency of dysphagia and regurgitation. Regarding the frequency of dysphagia, the value declined from 3.45 to 0.92 (*p* < 0.001)/1.00 (*p* = 0.02)/1.33 in successfully treated patients (six months after the 1st/2nd/3rd session) and for regurgitation from 2.85 to 0.35 (*p* < 0.001)/1.00 (*p* < 0.01)/0.67. Patients with recurrence needed in total a mean of 2.35 sessions (range 2-4) to achieve symptom control.

No bleeding occurred in the treatment of recurrences which means that the rate of bleeding was significantly lower in the retreatment group compared to the initial intervention group (*p* = 0.04). Also, there was no other severe adverse event in the treatment of recurrences.

## 4. Discussion

The strength of this study is the large cohort of patients and the follow-up over a median of 41 months (range 7-74). Besides the study of Huberty et al. [13], this is one of the largest prospectively documented cohort of patients with symptomatic ZD treated by a flexible endoscopic approach. Due to the long-term follow-up period, late recurrences were also included. Of course, there might be patients that are lost to follow-up so that recurrences are not detected. But this problem is similar to other interventional studies concerning ZD. The recurrence rate of 17% is comparable to the results of other studies with flexible endoscopy [6].

In surgical transcervical series, the reported recurrence rate varies widely, depending on the surgical technique, from 1.9% for open suspension [14] to 21% for invagination of the diverticulum [1].

In our study, patients who developed recurrence could be divided into two major groups. In group one (9%), after an initial distinct improvement of symptoms, recurrence occurred early within a few months after treatment. In the second group (8%), the patients were already in remission before they developed recurrent symptoms (median 16 months, range 8-26). A reason for the early recurrences might be an incomplete initial dissection of the cricopharyngeal muscle in the first session. Probably, the myotomy for those patients was not wide or long enough. An incomplete myotomy of the upper esophageal sphincter has already been discussed in the literature as a possible cause of recurrence [7, 15]. Why other patients, after an initially successful treatment with good symptom control, suffer a recurrence after a longer period of time cannot be conclusively explained at present.

There were no severe adverse events. In contrast, surgical approaches have a low but relevant number of severe adverse events such as mediastinitis or permanent palsy of the recurrent laryngeal nerve. Moreover, surgical meta-analysis shows a small number of therapy-related deaths [1, 14]. Also, in approaches with rigid endoscopes, some major adverse events, such as abscesses requiring external drainage, occurred [16].

Although the rate of recurrence in our cohort is slightly higher, this study has shown that treatment can be easily repeated with a high success rate. Repetition of a surgical treatment might be more difficult and challenging due to scarring tissue. In our cohort, retreatment was technically feasible, and in one patient, retreatment was performed four times. Retreatment of those patients was not associated with a higher rate of adverse events, and the rate of bleeding was even significantly lower. Dissection of the scar and remaining muscle tissue with flexible endoscopy is technically not more challenging than the initial treatment. Patients with recurrence could achieve the same control of their symptoms as patients without recurrence. Eventually, even patients who needed several sessions (mean 2.35 sessions) could be treated successfully.

Tunneling myotomy has been reported recently; however, further studies are needed to clarify its long-term effectiveness [17, 18]. It is also unclear if a tunneled myotomy could be repeated in case of recurrence.

A limitation of this study is that there is no direct comparison to other therapeutic methods, especially a transcervical surgical approach. A randomized study with different treatment paths would be able to compare the rate of recurrence and adverse events.

## 5. Conclusion

Symptomatic ZD can be controlled with endoscopic treatment using the sb knife jr in a safe and effective way. Patients with recurrence can be retreated without an increased risk of adverse events and with a high success rate. Patients with recurrence can ultimately achieve the same long-term symptom control as treatment-naïve patients.

## Figures and Tables

**Table 1:** Content of the questionnaire.

	0	1	2	3	4
Recorded symptoms					
Frequency of dysphagia	Never	<1/mth	<1/wk	>1/wk	Permanent
Dysphagia score	No dysph.	Solid food	Soft food	Fluids	Aphagia^∗^
Odynophagia	Never	<1/mth	<1/wk	>1/wk	Permanent
Regurgitation	Never	<1/mth	<1/wk	>1/wk	Permanent
Halitosis	Never	<1/mth	<1/wk	>1/wk	Permanent
Vomiting	Never	<1/mth	<1/wk	>1/wk	Permanent
Nocturnal awakening	Never	<1/mth	<1/wk	>1/wk	Permanent
Chronic cough	Never	<1/mth	<1/wk	>1/wk	Permanent
Additional information					
General condition	Very good	Good	Satisfactory	Bad	Very bad
Duration of symptoms	Open answer				
Most compromising sympt.	Open answer				
Weight loss	Open answer				

^∗^Aphagia means the inability to swallow saliva. dysph.: dysphagia; sympt.: symptoms; wk: week; mth: month.

**Table 2:** Demographic and clinical characteristics.

Total number of patients	100
Median age	71 (42-92)
Sex (female/male)	36/64
Median diverticular size (mm)	20 (10-45)
Total number of interventions	126
Number of the modified DISR technique	60 (47.6%)
Median follow-up (months)	41 (7-74)
Median body mass index (kg/m^2^)	26.1 (17.3-38.0)
Mean weight loss (kg)	2.3 (0-20)

Values express absolute numbers with (range).

**Table 3:** Frequency of symptoms of all patients before, one month, and six months after the initial treatment (mean value).

	Before	1 month	6 months
	*n* = 100	*n* = 91	*n* = 90
Dysphagia	3.45	0.62^∗∗∗^	1.09^∗∗∗^
Odynophagia	1.45	0.22^∗∗∗^	0.36^∗∗∗^
Regurgitation	2.85	0.30^∗∗∗^	0.56^∗∗∗^
Halitosis	1.15	0.25^∗∗∗^	0.27^∗∗∗^
Vomiting	0.79	0.13^∗∗∗^	0.09^∗∗∗^
Nocturnal awakening	1.97	0.05^∗∗∗^	0.23^∗∗∗^
Dry cough	2.85	0.78^∗∗∗^	0.98^∗∗∗^

Values express the following frequency scores: 0: never, 1: <1x/month, 2: >1x/month, 3: >1x/week, and 4: permanent. *n* = number of evaluated questionnaires. ^∗∗∗^*t*-test significance *p* < 0.001 compared to the value prior to the first treatment.

**Table 4:** Descriptive characteristics before treatment stratified by no recurrence/recurrence.

	Nonrecurrence	Recurrence	*p* value
Total number of patients	83	17	
Median follow-up (months)	44 (7-74)	39 (7-69)	
Median age	71 (42-92)	73 (49-85)	0.90
Sex (female/male)	32/51	4/13	0.24
Median diverticular size (mm)	20 (10-45)	27.5 (20-40)	0.26
Median body mass index (kg/m^2^)	25.8 (17.3-38.0)	27.1 (17.5-34.5)	0.70
Mean weight loss (kg)	2.1 (0-20)	2.3 (0-20)	0.83

Values express absolute numbers with (range).

## Data Availability

The data on which this study is based have been deposited in the study secretariat of the Department of Internal Medicine III at the University Hospital Augsburg.

## References

[1] Colombo-Benkmann M., Unruh V., Kocher T., Krieglstein C., Senninger N. (2003). Modern treatment options for Zenker’s diverticulum: indications and results. *Zentralblatt für Chirurgie*.

[2] van Overbeek J. J. (2003). Pathogenesis and methods of treatment of Zenker’s diverticulum. *The Annals of Otology, Rhinology, and Laryngology*.

[3] Brueckner J., Schneider A., Messmann H., Gölder S. K. (2016). Long-term symptomatic control of Zenker diverticulum by flexible endoscopic mucomyotomy with the hook knife and predisposing factors for clinical recurrence. *Scandinavian Journal of Gastroenterology*.

[4] Ishioka S., Sakai P., Maluf Filho F., Melo J. M. (1995). Endoscopic incision of Zenker’s diverticula. *Endoscopy*.

[5] Mulder C. J., den Hartog G., Robijn R. J., Thies J. E. (1995). Flexible endoscopic treatment of Zenker’s diverticulum: a new approach. *Endoscopy*.

[6] Ishaq S., Hassan C., Antonello A. (2016). Flexible endoscopic treatment for Zenker’s diverticulum: a systematic review and meta-analysis. *Gastrointestinal Endoscopy*.

[7] Feussner H. (2011). Zenker’s diverticulum: pro operation. *Chirurg*.

[8] Gölder S. K., Brueckner J., Ebigbo A., Messmann H. (2018). Double incision and snare resection in symptomatic Zenker’s diverticulum: a modification of the stag beetle knife technique. *Endoscopy*.

[9] Cotton P. B., Eisen G. M., Aabakken L. (2010). A lexicon for endoscopic adverse events: report of an ASGE workshop. *Gastrointestinal Endoscopy*.

[10] Goelder S. K., Brueckner J., Messmann H. (2016). Endoscopic treatment of Zenker’s diverticulum with the stag beetle knife (sb knife) - feasibility and follow-up. *Scandinavian Journal of Gastroenterology*.

[11] Dakkak M., Bennett J. R. (1992). A new dysphagia score with objective validation. *Journal of Clinical Gastroenterology*.

[12] Brombart M. M. (1980). *Radiologie des Verdauungstraktes: Funktionelle Untersuchung und Diagnostik*.

[13] Huberty V., el Bacha S., Blero D., le Moine O., Hassid S., Devière J. (2013). Endoscopic treatment for Zenker’s diverticulum: long-term results (with video). *Gastrointestinal Endoscopy*.

[14] Verdonck J., Morton R. P. (2015). Systematic review on treatment of Zenker’s diverticulum. *European Archives of Oto-Rhino-Laryngology*.

[15] Costamagna G., Iacopini F., Bizzotto A. (2016). Prognostic variables for the clinical success of flexible endoscopic septotomy of Zenker’s diverticulum. *Gastrointestinal Endoscopy*.

[16] Wilken R., Whited C., Scher R. L. (2015). Endoscopic staple diverticulostomy for Zenker’s diverticulum: review of experience in 337 cases. *The Annals of Otology, Rhinology, and Laryngology*.

[17] Yang J., Zeng X., Yuan X. (2019). An international study on the use of peroral endoscopic myotomy (POEM) in the management of esophageal diverticula: the first multicenter D-POEM experience. *Endoscopy*.

[18] Li Q. L., Chen W. F., Zhang X. C. (2016). Submucosal tunneling endoscopic septum division: a novel technique for treating Zenker’s diverticulum. *Gastroenterology*.

